# Diagnostic Limitations of Hemoglobin A1c in the Setting of Compound Hemoglobinopathy: A Case Report of Sickle Cell Disease, Alpha Thalassemia, and Occult Diabetes

**DOI:** 10.7759/cureus.101623

**Published:** 2026-01-15

**Authors:** Katie Toperzer, Anthony Moon

**Affiliations:** 1 College of Osteopathic Medicine, Nova Southeastern University Dr. Kiran C. Patel College of Osteopathic Medicine, Clearwater, USA; 2 Family Medicine, Lakeland Regional Health, Lakeland, USA

**Keywords:** alpha thalassemia, anemia, diabetic ketoacidosis, fructosamine, hba1c, hemoglobinopathy, hyperglycemia, sickle cell disease

## Abstract

This case report illustrates the diagnostic limitations of hemoglobin A1c (HbA1c) in patients with coexisting hemoglobinopathies. Sickle cell disease (SCD) and alpha thalassemia are inherited disorders characterized by chronic anemia and altered red blood cell turnover, both of which can significantly interfere with the accuracy of HbA1c measurements. In such cases, HbA1c may appear falsely low, potentially delaying the diagnosis and treatment of diabetes mellitus. We describe a 63-year-old female with previously undiagnosed SCD and alpha thalassemia whose longstanding hyperglycemia and diabetic complications were masked by inappropriately low HbA1c values. This report emphasizes the importance of recognizing the limitations of HbA1c in vulnerable populations and highlights the need for alternative glycemic markers, such as fructosamine, in patients with hemoglobinopathies to ensure accurate diagnosis and monitoring of diabetes mellitus.

## Introduction

Hemoglobin A1c (HbA1c) is a widely used mechanism to diagnose and manage diabetes mellitus. HbA1c reflects chronic glycemia, quantifying the average blood glucose levels of patients over a two- to three-month period [1]. Its convenience, standardization, and prognostic value in long-term complications have made HbA1c a foundational biomarker in diabetes care. However, in vulnerable populations, such as those with underlying hemoglobinopathies like sickle cell disease (SCD) or thalassemia, HbA1c may yield misleading values due to altered red blood cell turnover and hemoglobin structure, complicating the interpretation of glycemic control [2].

SCD is an inherited hemoglobinopathy characterized by the presence of hemoglobin S (HbS), which polymerizes under deoxygenated conditions, causing red blood cells to assume a sickled shape [3]. These abnormally shaped cells are prone to hemolysis and have a significantly shortened lifespan, leading to chronic hemolytic anemia and vaso-occlusive complications [4]. Even in the absence of acute sickle cell crises, patients with SCD may experience subclinical hemolysis and baseline anemia, which can interfere with diagnostic tools that rely on red blood cell survival.

Alpha thalassemia is an inherited blood disorder characterized by reduced or absent synthesis of alpha-globin chains, leading to the formation of abnormal hemoglobin and microcytic anemia [5]. The clinical severity of alpha thalassemia varies depending on the number of gene deletions, ranging from asymptomatic carrier states to hemolytic anemia [5]. In affected individuals, the altered hemoglobin composition and chronic anemia can influence the reliability of laboratory tests that depend on normal erythrocyte lifespan and hemoglobin structure. In conditions such as hemolytic anemias or hemoglobinopathies, shortened RBC survival reduces the time hemoglobin is exposed to circulating glucose, resulting in lower-than-expected HbA1c levels despite chronic hyperglycemia [6]. This principle also applies to other situations that affect RBC turnover, including recent transfusion or increased erythropoiesis.

Here, we report a case of disproportionately low HbA1c in the setting of new-onset insulin resistance, SCD, and alpha thalassemia. This case places emphasis on the limitations of HbA1c as a diagnostic and monitoring tool in patients with underlying hemoglobinopathies and highlights the importance of integrating alternative glycemic assessments in select populations.

## Case presentation

A 63-year-old African American female with previously unrecognized hemoglobinopathies presented to the emergency department with a one-week history of polyuria, polydipsia, and vaginal candidiasis. The patient reported a history of sickle cell trait that had not been evaluated since age 16. Initial laboratory evaluation revealed a blood glucose level of 609 mg/dL, and she was diagnosed with diabetic ketoacidosis (DKA). The patient was admitted and treated with intravenous fluids and continuous insulin infusion. On admission, extended labs revealed an HbA1c level of 4.8%.

**Figure 1:**
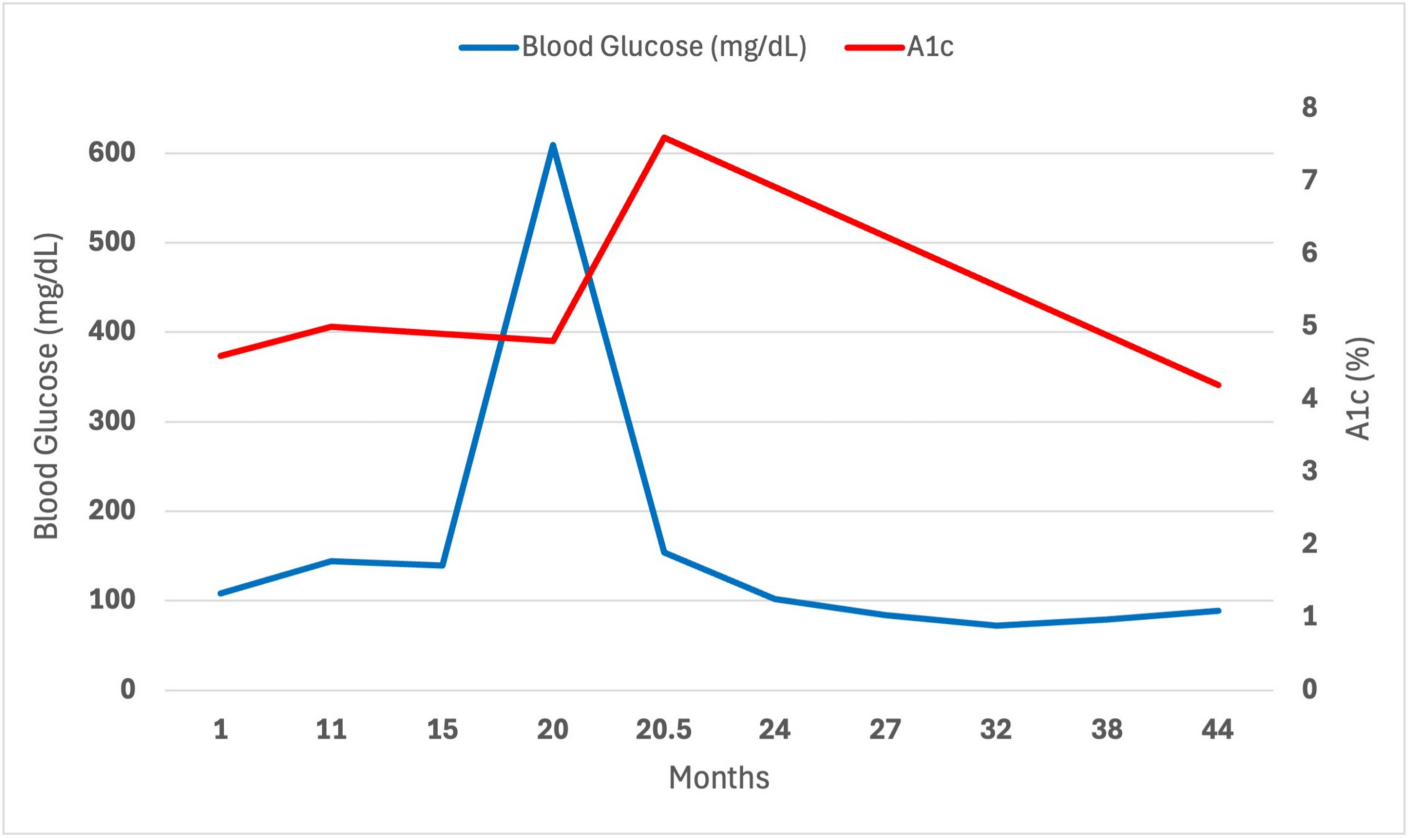
Blood glucose and HbA1c trends prior to and following diabetes mellitus diagnosis. Despite episodes of severe hyperglycemia, HbA1c values remain disproportionately low on the graph, demonstrating a significant discordance between short-term glucose measurements and long-term glycemic markers. Such discordance raises concern for unreliable assessment of glycemic control using HbA1c alone. HbA1c: hemoglobin A1c.

At outpatient follow-up, she remained hyperglycemic, with a blood glucose level of 287 mg/dL (Table 1). The patient was initiated on long-acting insulin and tirzepatide, with glycemic monitoring via a continuous glucose monitor. At that time, her HbA1c was 7.6% (Table 1). Retrospective review of prior laboratory data revealed persistently elevated fasting blood glucose values ranging from 108 mg/dL to 144 mg/dL, with unexpectedly low HbA1c values between 4.6% and 5.0% (Table 1 and Figure 1).

**Table 1:** Blood test results performed before hospital admission, at hospital admission, and at follow-up office visits. MCV: mean corpuscular volume; MCH: mean corpuscular hemoglobin; MCHC: mean corpuscular hemoglobin concentration; RDW: red cell distribution width; AST: aspartate aminotransferase.

Test	Reference range	1 year pre-admission	Hospital admission	2-week hospital follow-up	1-year follow-up	2-year follow-up
Blood glucose	70-99 mg/dL	144	609	287	72	89
A1c	4.0-6.0%	5.0	4.8	7.6		4.2
Fructosamine	200-285 µmol/L				253	
WBC	3.4-10.8 x10^3^/µL	10.1	12.0	9.8	8.6	8.9
RBC	3.77-5.28 x10^6^/µL	5.4	6.1	4.6	5.1	5.1
Hemoglobin	11.1-15.9 g/dL	10.7	12.4	9.4	10.2	10.2
Hematocrit	34.0-46.6%	36.1	36.4	31.8	34.2	29.8
MCV	79-97 fL	66.7	59.8	69.1	66.8	58.7
MCH	26.6-33.0 pg	19.8	20.4	20.4	19.9	20.1
MCHC	31.5-35.7 g/dL	29.6	34.1	29.6	29.8	34.2
RDW	11.7-15.4%	21.0	20.0	19.9	19.7	18.8
Platelets	150-450 x10^3^/µL	221	209	245	212	215
Total bilirubin	0.2–1.2 mg/dL	0.9	1.2	1.4		
Direct bilirubin	0.0–0.3 mg/dL		0.3			
Indirect bilirubin	0.2–0.9 mg/dL		0.9			
AST	10–40 U/L	15	70	16		
Total protein	6.0–8.3 g/dL			6.9	7.0	7.2
Total albumin	3.5–5.0 g/dL			4.3	4.4	4.4

Given the discordance between HbA1c and blood glucose, endocrinology and hematology were consulted. The patient reported progressive visual changes over several months and chronic peripheral neuropathy over the course of several years, not previously mentioned at primary care appointments, raising concern for longstanding, undiagnosed diabetes mellitus. Further evaluation by hematology revealed the patient to have a HbS of 49.1% and a hemoglobin F (HbF) of 3.1% via high-performance liquid chromatography (HPLC) and capillary electrophoresis, consistent with SCD, formerly misdiagnosed as sickle cell trait. Hematology also ordered PCR testing, which revealed the patient to have two alpha-globin gene deletions consistent with alpha thalassemia minor.

Over the subsequent two years of follow-up, serial HbA1c measurements remained consistently below 4.2% despite sustained glycemic control (Table 1). Due to the unreliability of HbA1c in this context, fructosamine was utilized to monitor glycemic trends. Fructosamine levels remained stable, albeit at the upper-normal range, from 253 µmol/L to 263 µmol/L, confirming adequate, but not ideal, glycemic management (Table 1). Additionally, the patient was found to have chronic anemia, initially identified incidentally approximately eight months prior to her diabetes diagnosis. Workup for chronic anemia had begun prior to DKA presentation; however, it remained incomplete. Her hemoglobin levels have remained persistently low, ranging from 9.4 g/dL to 10.7 g/dL over several years (Table 1).

## Discussion

This report addresses the discrepancies in HbA1c and hemoglobinopathies affecting red blood cell lifespan in the timely diagnosis of new-onset diabetes mellitus. Discrepancies between HbA1c and direct glucose measurements can be clinically significant, especially in cases of new-onset diabetes, where misinterpretation may delay diagnosis or obscure disease severity. Coexisting SCD and alpha thalassemia influence erythrocyte lifespan and may interfere with laboratory methods used to quantify HbA1c, potentially resulting in falsely low values.

Laboratory evaluation demonstrated only minimal biochemical evidence of hemolysis. Total bilirubin peaked at 1.4 mg/dL with indirect bilirubin at the upper limit of normal (0.9 mg/dL) and normal direct bilirubin, consistent with mild or compensated hemolysis (Table 1). Aspartate aminotransferase (AST) was largely within reference range, with a single transient elevation to 70 U/L, which is nonspecific and may reflect extrahepatic sources (Table 1) [7]. Notably, no marked or sustained hyperbilirubinemia was observed, arguing against an acute hemolytic process. However, the absence of additional hemolysis markers, such as lactate dehydrogenase, haptoglobin, and reticulocyte count, limits definitive quantification of hemolytic burden. Therefore, while these findings suggest only low-grade hemolysis, clinically meaningful reductions in erythrocyte lifespan cannot be excluded.

In this case, inpatient laboratory testing at the time of DKA admission demonstrated an HbA1c of 4.8% (Table 1). However, follow-up testing performed approximately two weeks after hospitalization revealed an elevated HbA1c of 7.6% (Table 1). Review of the patient’s hemoglobin and other markers of anemia revealed no evidence of significant blood loss or hemolysis that could account for such a rapid rise in HbA1c over a short interval. Notably, the two sets of laboratories were obtained at different institutions using assays with potentially differing methodologies, which may have contributed to variability. The magnitude of this discordance is unlikely to be explained by physiologic changes alone and strongly suggests laboratory measurement error as the primary cause; however, assay interference secondary to underlying hemoglobinopathies cannot be excluded.

HbA1c values may be misleading in patients with hemoglobinopathies due to both biologic and analytic factors. In SCD, chronic hemolysis shortens RBC lifespan, reducing the time hemoglobin is available for glycation and thereby producing falsely low HbA1c values despite sustained hyperglycemia [8]. Alpha thalassemia, in contrast, has been variably associated with false elevations or reductions in HbA1c depending on erythrocyte survival and assay methodology [9]. Previous reports of alpha thalassemia have shown variant hemoglobin chains elute in the same fraction as HbA1c [10-12]. Severe microcytosis, as observed in this case (mean corpuscular volume = 58-69 fL), may further impair accurate quantification of glycated hemoglobin in certain assays. When alpha thalassemia coexists with a highly hemolytic disorder such as SCD, the effect of reduced RBC survival likely predominates, resulting in an overall falsely low HbA1c [8]. These competing mechanisms emphasize the limitations of HbA1c interpretation in complex hemoglobinopathies and support the use of alternative glycemic markers.

Fructosamine is a stable ketoamine formed from the glycation reaction of glucose with various plasma proteins, primarily albumin [13]. Increases in blood glucose, as seen in diabetes, increase the level of fructosamine, allowing it to be an alternative biomarker for glycemic control. Due to the nature of plasma proteins, fructosamine can be monitored over a shorter span of two to three weeks compared to red blood cells at 90 to 120 days [14]. Unlike HbA1c, fructosamine levels are measured independent of hemoglobin variants and erythrocyte lifespan, making it particularly useful in patients with hemoglobinopathies [13].

Interpretation of fructosamine levels in this case must be considered in the context of known assay limitations. Due to the reliance on circulating serum proteins, predominantly albumin, using fructosamine may be unreliable in conditions associated with hypoalbuminemia (typically <3.0 g/dL), such as cirrhosis or nephrotic syndrome, or in states of elevated total protein due to increased immunoglobulins [7]. In this patient, however, serum albumin and total protein levels remained within normal reference ranges, supporting the validity of fructosamine as an indicator of short-term glycemic exposure. Nonetheless, fructosamine does not account for alterations in protein turnover or erythrocyte-independent metabolic variability, and results should be interpreted in conjunction with the broader clinical context.

The novelty of this case serves as an important reminder that HbA1c is not universally reliable, particularly in patients with compound hemoglobinopathies or chronic anemia. Alternative methods, such as fructosamine or continuous glucose monitoring, may provide more accurate assessments of glycemia in these populations [15]. Timely recognition of these discrepancies is essential to avoid delayed diagnosis, inappropriate treatment decisions, and progression of diabetic complications.

## Conclusions

This case illustrates the critical limitations of HbA1c in accurately diagnosing and monitoring diabetes in niche patients with underlying hemoglobinopathies such as SCD and alpha thalassemia. In such cases, reliance on HbA1c alone can mask the presence and severity of hyperglycemia, potentially delaying diagnosis and appropriate treatment. Alternative glycemic biomarkers, such as fructosamine, should be considered when HbA1c values are discordant with clinical or biochemical evidence of dysglycemia. This most commonly occurs in conditions affecting erythrocyte lifespan or hemoglobin structure, including hemolytic anemias, hemoglobinopathies, recent blood loss or transfusion, or increased erythropoiesis, where HbA1c may underestimate true glycemic exposure.

In patients presenting with clinical or biochemical features suggestive of hyperglycemia but unexpectedly low HbA1c, it is important to consider conditions that affect red blood cell lifespan or hemoglobin structure, such as hemoglobinopathies or hemolytic anemia. Discordance between HbA1c and plasma glucose can be quantitatively defined. Labs revealing elevated blood glucose can be indicative of discordance, as such a mismatch suggests that HbA1c may be underestimating true glycemic exposure due to shortened erythrocyte survival or abnormal hemoglobin glycation. Identifying these cases is critical, as relying solely on HbA1c could lead to delayed diagnosis or mismanagement of diabetes. Continuous glucose monitoring (CGM) provides a practical, real-time alternative because it measures interstitial glucose independent of erythrocyte lifespan, capturing glycemic variability and hyperglycemic excursions that HbA1c may miss. By combining threshold-based evaluation with CGM or short-term biomarkers like fructosamine, clinicians can obtain a more accurate assessment of glycemic control, guiding timely and appropriate management in patients where HbA1c alone is unreliable.

## References

[1] American Diabetes Association (2010). Diagnosis and classification of diabetes mellitus. Diabetes Care.

[2] Klonoff DC (2020). Hemoglobinopathies and hemoglobin A1c in diabetes mellitus. J Diabetes Sci Technol.

[3] Elendu C, Amaechi DC, Alakwe-Ojimba CE (2023). Understanding sickle cell disease: causes, symptoms, and treatment options. Medicine (Baltimore).

[4] Steinberg MH (2008). Sickle cell anemia, the first molecular disease: overview of molecular etiology, pathophysiology, and therapeutic approaches. ScientificWorldJournal.

[5] Farashi S, Harteveld CL (2018). Molecular basis of α-thalassemia. Blood Cells Mol Dis.

[6] Kutter D, Thoma J (2006). Hereditary spherocytosis and other hemolytic anomalies distort diabetic control by glycated hemoglobin. Clin Lab.

[7] Armbruster DA (1987). Fructosamine: structure, analysis, and clinical usefulness. Clin Chem.

[8] Li M, Ge S, Shu X, Wu X, Liu H, Xu A, Ji L (2024). Interference of hemoglobin variants with HbA1c measurements by six commonly used HbA1c methods. Lab Med.

[9] Xu A, Ji L, Chen W, Xia Y, Zhou Y (2016). Effects of α-thalassemia on HbA1c measurement. J Clin Lab Anal.

[10] Xu A, Sun J, Li J, Chen W, Zheng R, Han Z, Ji L (2019). Hb I: a α-globin chain variant causing unexpected HbA1c results. J Clin Lab Anal.

[11] Singha K, Fucharoen G, Chaibunruang A, Netnee P, Fucharoen S (2012). A spurious haemoglobin A1c result associated with double heterozygote for haemoglobin Raleigh (β1[NA1]Val → Ala) and α+-thalassaemia. Ann Clin Biochem.

[12] Gao W, Jin Y, Huang Y, Tang H (2022). Discordantly high glycated hemoglobin might assist in diagnosing α-thalassemia, but not diabetes: a case report. J Diabetes Investig.

[13] Gounden V, Anastasopoulou C, Zubair M, Jialal I (2025). Clinical utility of fructosamine and glycated albumin. StatPearls.

[14] Danese E, Montagnana M, Nouvenne A, Lippi G (2015). Advantages and pitfalls of fructosamine and glycated albumin in the diagnosis and treatment of diabetes. J Diabetes Sci Technol.

[15] Chehregosha H, Khamseh ME, Malek M, Hosseinpanah F, Ismail-Beigi F (2019). A view beyond HbA1c: role of continuous glucose monitoring. Diabetes Ther.

